# tRF and gastric cancer: molecular mechanism exploration and novel strategies for precision diagnosis and therapy

**DOI:** 10.1186/s12967-026-08474-7

**Published:** 2026-06-16

**Authors:** Hongping Jia, Yiyao Duan, Yun Huang, Jun Hu, Xirui Fan, Rui Gong, Ningyan Zhang, Rong Qin, Hui Wang

**Affiliations:** 1 Department of Gastroenterology, Yan’ an Hospital Affiliated to Kunming Medical University, Kunming, Yunnan 650051 China; 2 Key Laboratory of Tumor Immunological Prevention and Treatment of Yunnan Province, Kunming, 650051 China; 3 Calmette Hospital Affiliated to Kunming Medical University, Kunming, Yunnan, 650034 China

**Keywords:** tRNA-derived fragments (tRFs), Gastric cancer, Molecular mechanisms, Biomarkers, Targeted therapy

## Abstract

**Background:**

Gastric cancer (GC) remains a leading cause of cancer-related mortality, and current diagnostic biomarkers lack sufficient sensitivity and specificity. tRNA-derived fragments (tRFs), an emerging class of non-coding RNAs, have garnered attention for their stability and regulatory roles in carcinogenesis.

**Main body:**

This review adopts a comparative perspective to evaluate the distinctive roles of tRFs in GC relative to other non-coding RNAs, particularly microRNAs (miRNAs). We summarize tRF biogenesis and classification, highlighting their unique mechanistic repertoire that encompasses both AGO2-dependent gene silencing and non-canonical protein interactions. Dysregulated tRF profiles in patient samples reveal promising diagnostic candidates (e.g., tRF-23-Q99P9P9NDD, tRF-17-18VBY9M) that demonstrate superior performance to conventional markers. However, we critically examine translational barriers including functional heterogeneity, detection challenges, and the absence of registered clinical trials. We also dissect sources of contradictory findings and propose standardized frameworks for future investigation.

**Conclusions:**

tRFs represent functionally versatile regulators with distinct advantages over miRNAs in diagnostic applications. Their clinical translation requires overcoming methodological standardization gaps and evidence thresholds through interdisciplinary efforts integrating multi-omics profiling, advanced delivery systems, and rigorous clinical validation.

## Introduction

GC is a prevalent malignant tumor worldwide, exhibiting substantial geographic heterogeneity in both incidence and mortality rates [1]. Epidemiological data indicate that East Asia bears over 60% of the global GC burden, with Helicobacter pylori infection, high-salt diet, and genetic susceptibility identified as the predominant risk factors [2]. China, a region with a high incidence of GC, accounts for 44.0% of global new cases and 48.6% of deaths worldwide. Notably, GC ranks as the third most common malignancy in China in terms of both incidence and mortality rates [3]. Although developed countries have achieved a consistent decline in GC incidence through Helicobacter pylori eradication programs, widespread endoscopic screening, and the adoption of multidisciplinary treatment approaches [4–6], the emerging global trend of early-onset gastric cancer (EOGC, < 50 years) underscores the urgent need to develop precision prevention and treatment strategies for this novel high-risk population.

Gastroscopy has been established as the gold standard for GC diagnosis owing to its direct visualization capability. However, its application in population-based screening remains substantially limited by inherent drawbacks, including procedure-related patient discomfort, high medical costs, and significant operator dependence [7, 8]. Furthermore, tumor biomarkers serve dual roles in GC management, functioning both as early screening tools and for therapeutic monitoring and prognostic evaluation. Currently employed serum biomarkers (e.g., CA72-4, CEA, CA19-9) demonstrate significantly improved sensitivity (60–70%) when utilized in combination strategies [9], thereby partially compensating for the limited sensitivity of individual markers (e.g., CA19-9 sensitivity of merely 25–40%) [10]. However, these protein-based biomarkers still exhibit substantial limitations [11–13] : (1) Limited tissue specificity, as elevated levels may also occur in benign conditions (e.g., gastritis, peptic ulcers); (2) Limited detection efficacy for early-stage disease (CA72-4 shows < 20% positivity rate in stage I GC); (3) Compromised monitoring utility due to tumor heterogeneity, for instance, diffuse-type gastric cancers typically demonstrate lower CEA expression levels than intestinal-type tumors. These limitations have catalyzed the exploration of novel molecular biomarkers, with non-coding RNAs (ncRNAs) emerging as a research focus due to their tissue-specific expression patterns and multidimensional regulatory functions in GC pathogenesis [14, 15].

tRFs, as emerging members of the ncRNA family, have garnered significant attention due to their high stability and diverse regulatory mechanisms [16–18]. However, amidst the expanding literature on tRFs in GC, a critical question remains inadequately addressed: what distinguishes tRFs from other well-characterized ncRNAs, particularly microRNAs (miRNAs), in terms of their biological properties, clinical potential, and translational challenges? Accumulating studies have demonstrated that tRFs drive GC invasion, metastasis, and immune microenvironment remodeling by binding to Argonaute (Ago) proteins to mediate gene silencing, modulating ribosome function, and activating the Toll-like receptor/nuclear factor kappa B (TLR/NF-κb) inflammatory pathway [19, 20]. These findings not only elucidate the multi-dimensional regulatory network of tRFs in GC but also highlight their translational potential as liquid biopsy biomarkers and therapeutic targets.

This review adopts a comparative perspective and systematically examines the unique role of tRFs in GC, with the aim of: (1) delineate the unique biological features that differentiate tRFs from miRNAs and other ncRNAs; (2) critically evaluate whether tRFs demonstrate superior diagnostic performance compared to conventional biomarkers (CEA, CA19-9, CA72-4) based on available head-to-head evidence; (3) assess the translational potential of tRF-based therapies in light of their mechanistic diversity; and (4) analyze the specific methodological and biological challenges that render tRF research particularly complex. By framing tRFs within this comparative context, we seek to provide a theoretical foundation for ncRNA-based precision medicine strategies in GC, thereby facilitating the translation of basic research into clinical practice.

For this narrative review, we conducted a literature search in PubMed and Web of Science (up to February 2026) using keywords related to tRFs and GC, prioritizing studies that provided mechanistic insights or comparative data with other biomarkers.

## Biological functions and structural features of tRFs

tRFs, a functionally important subclass of tRNA-derived small RNAs (tsRNAs), are generated through specific enzymatic cleavage of tRNAs by nucleases (e.g., ELAC2/RNase Z, RNase L, Dicer, and ANG) rather than random degradation [21, 22]. According to cleavage sites and genomic origins, tRFs can be systematically classified into distinct subtypes, including tRF-1s (derived from the 3′ end of tRNA precursors), tRF-5s/tRF-3s (cleaved from the 5′ or 3′ end of mature tRNAs, respectively), tiRNAs (tRNA halves), and i-tRFs (internal tRNA-derived fragments) [22–24] (Fig. 1A and 1B). Unlike tRF-3s and tRF-5s, tRF-1s are generally not loaded into RISC/AGO2 complexes and primarily function through other mechanisms. tRFs typically contain the oligonucleotide/oligosaccharide-binding (OB) fold, a conserved structural domain that mediates specific recognition of substrates such as carbohydrates, nucleic acids, and proteins. This molecular feature enables their involvement in critical biological processes, including genome stability maintenance, transcriptional regulation, translation quality control, and dynamic tRNA metabolism [25–27]. It is worth noting that tRFs represent a major subclass of tRNA-derived small RNAs (tsRNAs), distinct from other tsRNA types such as stress-induced tiRNAs (tRNA halves) primarily by their constitutive rather than stress-responsive biogenesis and their broader spectrum of physiological functions. While tiRNAs are typically generated under stress conditions (e.g., hypoxia, nutrient deprivation) via angiogenin cleavage, tRFs are produced constitutively through specific enzymatic processing and participate in diverse regulatory processes under both physiological and pathological conditions.

**Fig. 1A:**
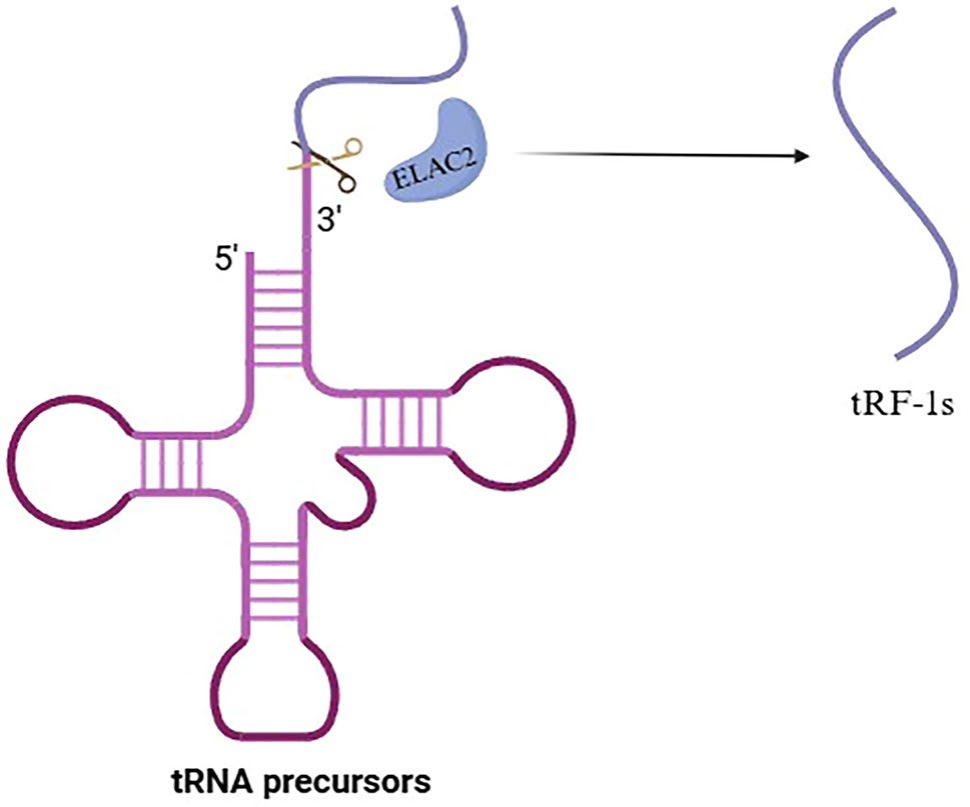
tRNA precursors by ELAC2 generates tRF-1s. Created in https://BioRender.com

**Fig. 1B:**
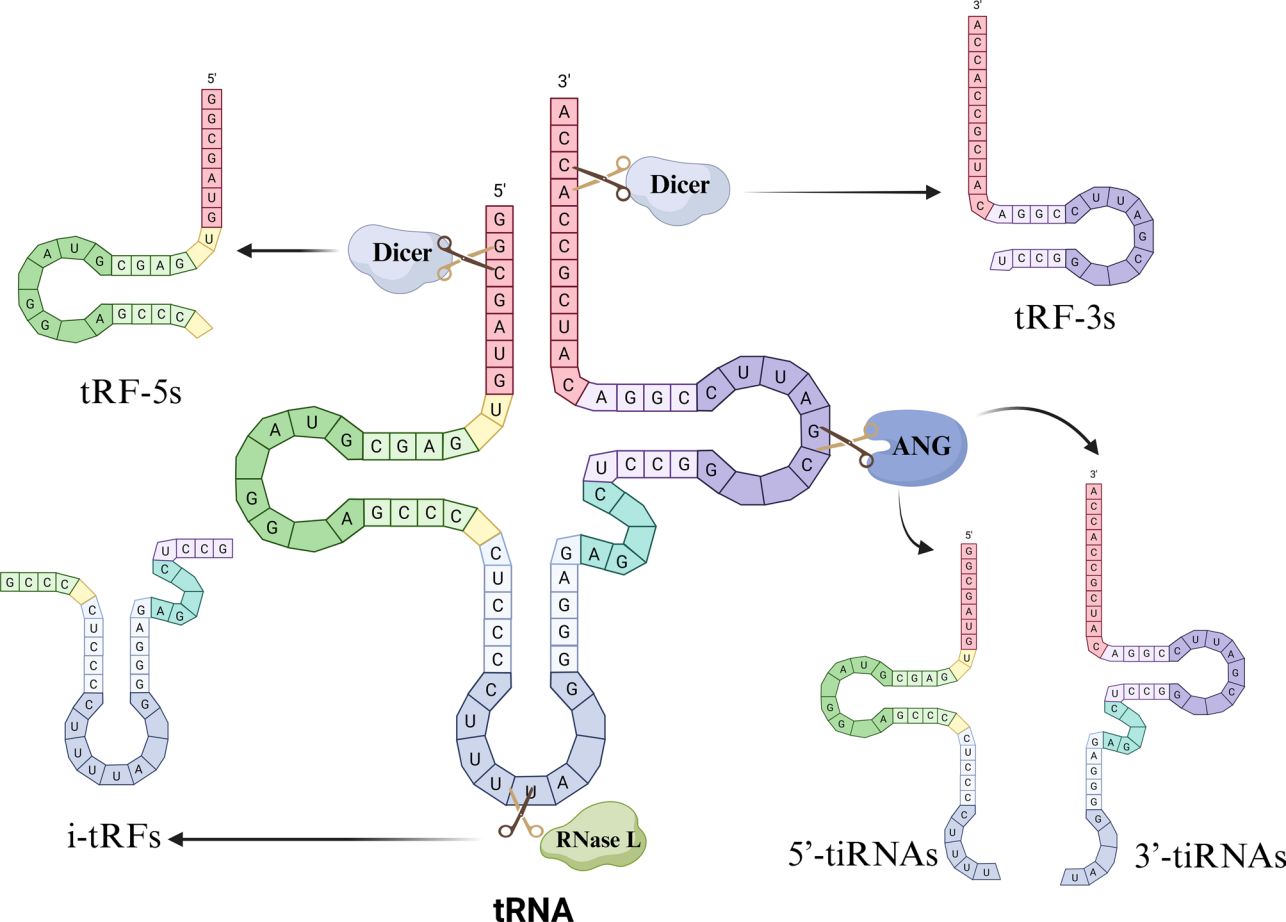
Cleavage of mature tRNA by Dicer, ANG, and RNase L produces functional tRFs including tRF-5s, tRF-3s, tiRNAs, and i-tRFs. Created in https://BioRender.com

**Fig. 2:**
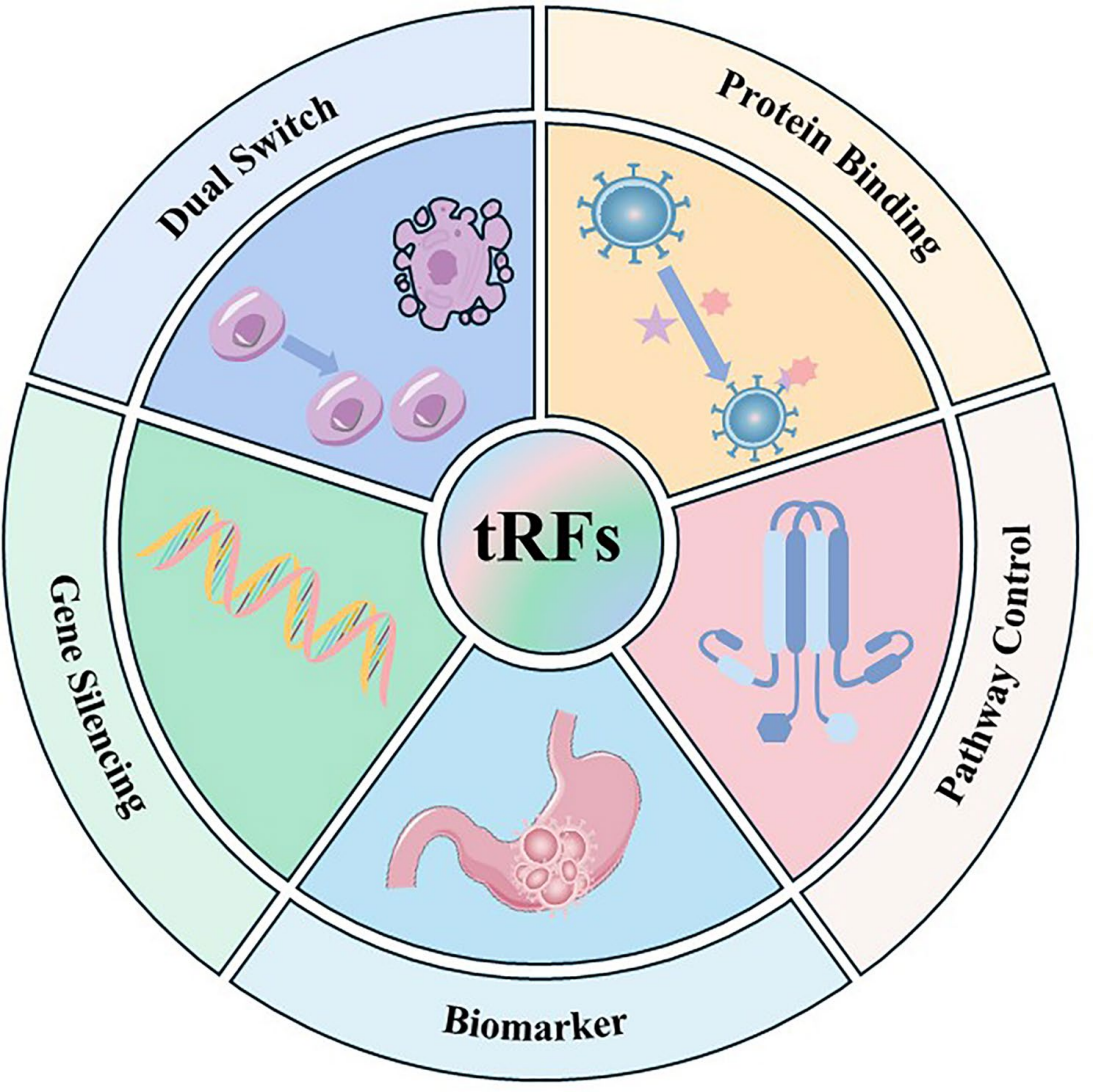
Summary of known functions of tRFs

tRFs exhibit multifaceted biological functions, including: (1) Gene Expression Regulation: tRFs modulate transcriptional and translational processes by directly binding to mRNA or competitively inhibiting RNA-binding proteins (RBPs) [28, 29]; (2) Cell Fate Determination: Certain tRFs induce apoptosis through activation of pro-apoptotic signaling pathways, while others promote cell survival by suppressing apoptosis-related proteins [30–32]; (3) Epigenetic Modulation and Viral Regulation: tRFs participate in chromatin remodeling, RNA interference, and viral replication cycles. For instance, they can influence viral life cycles by binding to reverse transcriptase [28, 33, 34]. Notably, tRFs exhibit tissue-specific expression patterns in cancer, with certain subtypes (e.g., tRF-5s) demonstrating aberrant overexpression in tumor tissues, potentially driving malignant progression through the activation of oncogenic signaling pathways [16, 35–38] (Fig. 2).

### tRFs in GC: expression and mechanisms

#### Dysregulated expression of tRFs

The aberrant expression of tRFs in GC patients demonstrates significant associations with tumor progression and clinical characteristics. Current research has identified multiple differentially expressed tRFs in plasma, serum, and tissue samples from GC patients, suggesting their potential involvement in regulating gastric carcinogenesis and disease progression [39–41]. RNA sequencing analysis revealed 21 specifically upregulated and 46 downregulated tRFs in the plasma of GC patients, demonstrating marked heterogeneity in tRF expression profiles associated with GC [42]. For example, tRF-33-P4R8YP9LON4VDP demonstrates specific upregulation in the plasma of GC patients, suggesting its potential involvement in pro-oncogenic signaling pathways [42]; notably, tRF-29-R9J8909NF5JP exhibits concomitant overexpression in both GC tissues and serum, with its postoperative serum levels showing significant reduction. Clinically relevant correlations were observed between elevated tRF-29-R9J8909NF5JP expression and poorly differentiated histology, advanced TNM staging, and lymph node metastasis [43, 44]; furthermore, tRF-17-18VBY9M demonstrates consistent upregulation in both serum and tissue samples, with receiver operating characteristic (ROC) curve analysis confirming its superior diagnostic performance compared to conventional biomarkers (CEA, CA19-9, and CA72-4) [45]. Conversely, low-abundance tRFs may exert tumor-suppressive effects through negative regulatory mechanisms. Notably, tRF-19-3L7L73JD demonstrates significantly reduced expression in preoperative plasma samples compared to both healthy controls and postoperative groups, suggesting its potential role in inhibiting tumor progression [46]; the i-tRF-AsnGTT exhibits significantly reduced expression in GC serum, with postoperative recovery of its levels. Its expression intensity shows a negative correlation with key clinicopathological parameters, including tumor differentiation grade and infiltration depth [47]; 3-tRF-Arg displays specific downregulation in GC serum and shows independent associations with early-stage tumor differentiation and lymph node metastasis, suggesting its potential role in early pathological progression [48]. Collectively, these findings underscore that tRF expression heterogeneity is closely linked to both the molecular regulatory networks driving GC progression and clinical staging parameters (Table 1).

**Table 1:** Summary of tRF expression and mechanism of action in gastric cancer

Name	Classification	Expression Trend	Mechanism and Function	References
tRF-33-P4R8YP9LON4VDP	tRF-3	Upregulated (plasma)	Silences STAT3 mRNA by binding to AGO2 protein, inhibits the STAT3 signaling pathway, reduces gastric cancer cell metastasis and induces apoptosis.	[42, 44]
tRF-Val	tRF-5s/tRF-3s	Upregulated (tissue)	Binds to EEF1A1 to promote its nuclear translocation, interacts with MDM2 to inhibit the p53 pathway, accelerating tumor progression.	[49]
tRF-Val-CAC-016	tRF-5s	Downregulated (tissue)	Targets CACNA1d to regulate the MAPK pathway, inhibiting gastric cancer cell proliferation.	[50]
tRF-Tyr	tRF-5s/tRF-3s	Downregulated (cells)	Competitively binds to hnRNPD protein, blocking its interaction with the c-Myc 3’UTR, inhibits the c-Myc/Bcl2/Bax pathway, suppresses proliferation and promotes apoptosis.	[20]
tRF-5026a	tRF-5s/tRF-3s	Downregulated (cells)	Inhibits the PTEN/PI3K/AKT pathway, reducing gastric cancer cell proliferative	[51]
tRF-23-Q99P9P9NDD	tRF-5s/tRF-3s	Upregulated (serum)	Significantly highly expressed in serum, distinguishes gastric cancer patients from gastritis patients, diagnostic efficacy superior to traditional markers (CEA, CA199).	[52]
tRF-17-18VBY9M	i-tRFs	Upregulated (serum/tissue)	Specifically upregulated in both serum and tissue, accurately discriminates early gastric cancer from benign lesions, ROC curve shows high diagnostic value.	[45]
tRF-19-3L7L73JD	tRF-5s/tRF-3s	Downregulated (preoperative plasma)	Low expression in preoperative plasma, increases postoperatively, may inhibit tumor progression through negative regulation.	[46]
i-tRF-AsnGTT	i-tRFs	Downregulated (serum)	Low expression in serum negatively correlates with tumor differentiation grade, levels recover postoperatively, suggesting tumor-suppressive role.	[47]
3-tRFArg	tRF-3s	Downregulated (serum)	Low expression in serum independently associated with early tumor differentiation and lymph node metastasis, may be involved in early pathological regulation.	[48]
tRF-24-6VR8K09LE9	tRF-5s/tRF-3s	Downregulated (tissue)	Inhibits the PI3K/AKT pathway to delay tumor progression, its low expression can serve as an early molecular warning marker for gastric cancer.	[53]

#### Mechanistic roles of tRFs in GC

Recent studies have revealed that tRFs exert multidimensional regulatory effects on GC pathogenesis by targeting key signaling pathways and molecular interaction networks. For instance, tRF-33-P4R8YP9LON4VDP has been shown to silence signal transducer and activator of transcription 3 (STAT3) mRNA by binding to AGO2 protein, thereby suppressing STAT3 pathway activity, which significantly reduces metastatic potential and induces apoptosis in GC cells [44]. Conversely, upregulated tRF-Val promotes nuclear translocation of Eukaryotic translation elongation factor 1 alpha 1(EEF1A1) through direct binding and subsequently interacts with Mouse double minute 2 homolog(MDM2) to inhibit the p53 downstream pathway, ultimately accelerating tumor progression [49]. Furthermore, downregulated tRF-Val-CAC-016 has been demonstrated to suppress GC cell proliferation by targeting CACNA1d to regulate the MAPK signaling pathway [50]. Similarly, the significantly downregulated nuclear tRF-Tyr competitively binds to heterogeneous nuclear ribonucleoprotein D(hnRNPD) protein, thereby blocking its interaction with the 3’UTR of c-Myc. This mechanism ultimately modulates the c-Myc/Bcl-2/Bax pathway, resulting in the inhibition of tumor proliferation and promotion of apoptosis [20]. These studies demonstrate that tRFs influence malignant phenotypes in GC through distinct mechanisms, including RNA silencing, protein interactions, and pathway regulation, with their functional diversity being closely associated with subcellular localization and target molecule selection.

**Fig. 3:**
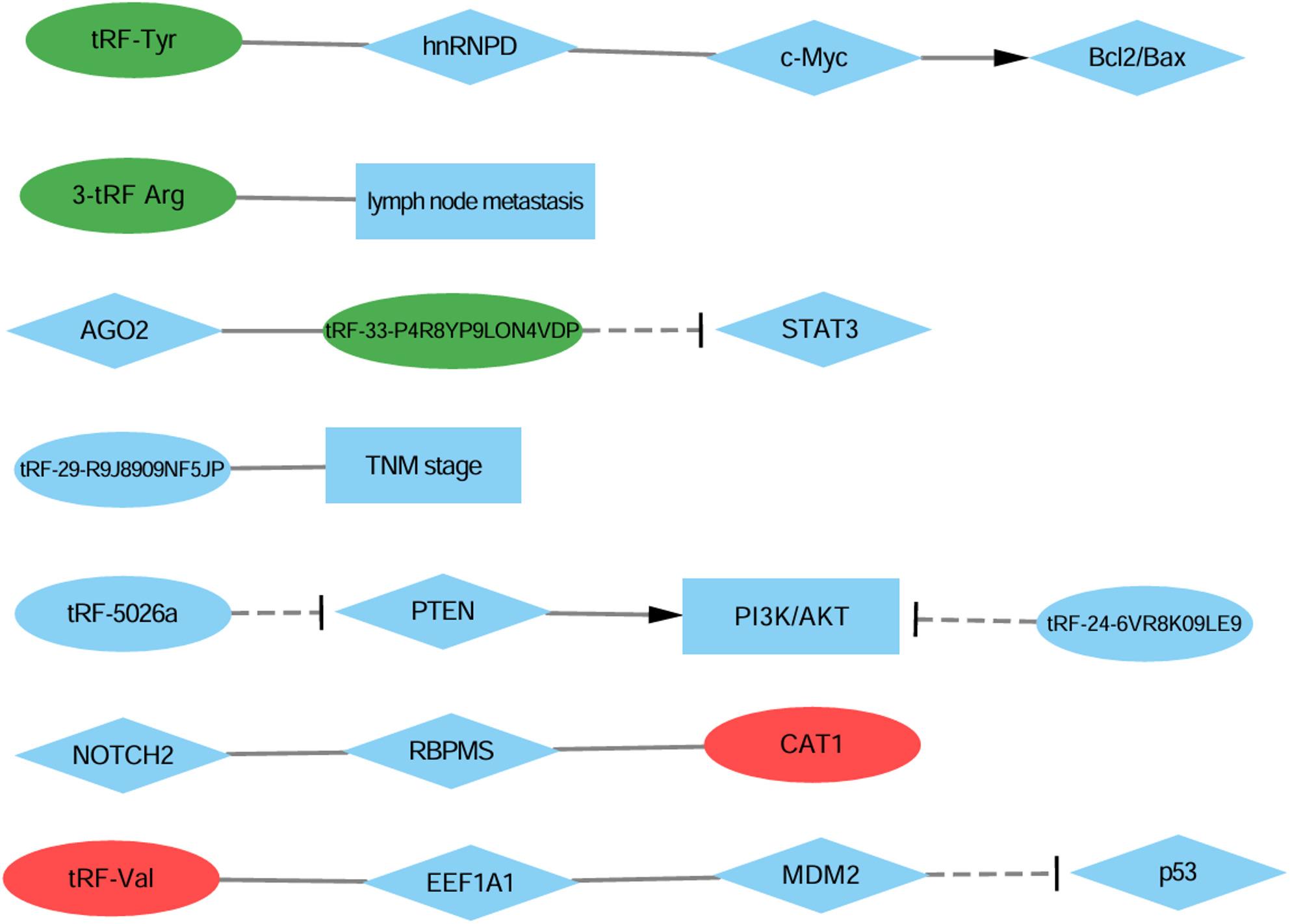
Schematic diagram of the mechanism of action of tRF in GC. Note: All symbols and lines follow standard conventions: red indicates upregulation, green indicates downregulation, solid arrows denote direct interactions, and dashed arrows represent indirect pathways

Moreover, tRFs exhibit dual regulatory roles in modulating GC cell proliferation and apoptosis, with their underlying mechanisms holding significant potential for clinical translation [54, 55]. For instance, tRF-5026a markedly suppresses cellular proliferative activity by inhibiting the PTEN/PI3K/AKT pathway [51]. By contrast, other pro-proliferative factors such as the spindle checkpoint protein TTK promote GC cell growth through activation of the Akt-mTOR signaling axis and correlate with poor patient prognosis [56]; whether tRFs directly modulate TTK expression or activity remains to be investigated. Notably, the molecular mechanisms of specific tRFs have unveiled novel therapeutic avenues for GC: The tumor-suppressive function of tRF-Tyr through modulation of the hnRNPD/c-Myc axis suggests its potential as a target for RNA interference therapy [20]; meanwhile, the interaction between tRF-Val and the EEF1A1/MDM2 complex indicates that targeting nuclear translocation processes may represent a viable intervention strategy [49] (Table 1). Future studies should focus on elucidating the subcellular localization specificity of tRFs, functional divergence among isoforms, and their crosstalk with the tumor microenvironment. Furthermore, integration of multi-omics data is required to decipher the regulatory networks between tRFs and other non-coding RNAs [49, 57–60], which will facilitate their clinical translation for GC diagnosis and precision therapy (Fig. 3).

Collectively, these mechanistic insights reveal both parallels and divergences between tRFs and the most extensively studied ncRNA family—microRNAs (miRNAs). Like miRNAs, certain tRFs (e.g., tRF-33-P4R8YP9LON4VDP) exert gene-silencing effects through AGO2-dependent pathways [44]. However, tRFs exhibit a broader mechanistic repertoire: they can directly bind to and modulate the function of RNA-binding proteins such as EEF1A1 [49] and hnRNPD [20]—interactions rarely reported for canonical miRNAs. This mechanistic diversity, coupled with their distinct biogenesis from mature tRNAs, positions tRFs as functionally versatile regulators that operate beyond the classical RNA interference paradigm.

Regarding subtype-function associations, preliminary patterns emerge from available data (summarized in Table 1). tRF-5s and tRF-3s (e.g., tRF-33-P4R8YP9LON4VDP, tRF-Val) frequently engage AGO2 or other RNA-binding proteins to modulate signaling pathways (STAT3, p53), with functions validated primarily in GC cell lines (AGS, MKN45, MGC803) and, in some cases, xenograft mouse models [42, 44, 49]. i-tRFs (e.g., i-tRF-AsnGTT, tRF-17-18VBY9M) have been implicated as diagnostic biomarkers in patient serum/tissue samples, though their precise molecular mechanisms remain less characterized [45, 47]. tRF-Tyr, a 5’-tRF, was shown to exert tumor-suppressive effects by binding hnRNPD in MGC803 and BGC823 cells, with validation in BALB/c nude mouse xenografts [20]. These observations, while preliminary, provide a foundation for future systematic mapping of tRF subtype-function relationships. As summarized (Table 2), these mechanistic differences position tRFs as a functionally distinct ncRNA family with unique advantages and challenges compared to miRNAs.

**Table 2:** Comparative features of tRFs and microRNAs (miRNAs) in gastric cancer

Feature	tRFs	miRNAs	References
Biogenesis	Derived from mature tRNAs or pre-tRNAs via cleavage by Dicer, ELAC2, angiogenin, etc.	Derived from hairpin precursors via Drosha and Dicer processing	[21–24]
Subtypes	Multiple subtypes: tRF-5s, tRF-3s, i-tRFs, tRF-1s, tiRNAs	Single major class, with minor sequence variants (isomiRs)	[22–24]
AGO2-dependent mechanism	Some tRFs (e.g., tRF-33) function through AGO2-mediated gene silencing	Canonically function through AGO2-containing RISC complex	[44]
AGO2-independent mechanism	Other tRFs (e.g., tRF-Val, tRF-Tyr) function through direct binding to non-AGO proteins such as EEF1A1 and hnRNPD	Rare; most miRNAs require AGO proteins for function	[20, 49]
Protein interactions	Diverse RBPs including AGO2, EEF1A1, hnRNPD	Primarily AGO proteins; rare interactions with other RBPs	[20, 44, 49]
Structural features	Contain OB-fold domain derived from parental tRNA	Lack conserved structural domains; function determined by seed sequence	[25–27]
Stability	High stability conferred by tRNA modifications (e.g., m⁵C, m⁷G, pseudouridine)	Relatively stable but lack tRNA-specific modification signatures	[16, 17]
Expression pattern	Tissue-specific; expression correlates with parental tRNA expression	Tissue-specific; regulated at transcription and processing levels	[61–63]
Diagnostic potential	Emerging biomarker in plasma/serum; some show superiority to CEA/CA19-9 (e.g., tRF-23, tRF-17)	Extensively studied as circulating biomarkers; several in clinical validation	[45, 52]
Therapeutic targeting	Preclinical stage; synthetic mimics/inhibitors under investigation	Multiple candidates in clinical trials (e.g., MRX34, cobomarsen)	[64]

The regulation of tRF expression exhibits remarkable complexity. In the model plant Arabidopsis thaliana, approximately 50% of tRNA genes demonstrate minimal expression (contributing to < 1% of total tRNA abundance), while a mere 12% of highly expressed genes account for 80% of the total tRNA pool. This observation suggests the existence of stringent hierarchical control in tRNA synthesis [61]; in mammals, although tRNA isodecoder genes exhibit significant expression heterogeneity across tissues, the anticodon pools within each tRNA family remain relatively stable. This suggests that tRNA expression regulation is finely tuned to maintain tissue-specific adaptability [62, 63]. These findings reveal a dynamic equilibrium between evolutionary conservation and species specificity in tRFs, providing a theoretical framework for understanding their functional heterogeneity in disease pathogenesis [65–67]. These mechanistic insights, together with the complex regulation of tRF expression discussed below, set the stage for understanding the challenges that lie ahead in translating tRF research.

### Clinical potential of tRFs in GC

#### The role of tRFs in early GC diagnosis

tRFs demonstrate significant potential as biomarkers for the early diagnosis of GC [43, 68, 69]. Moreover, their high sensitivity and specificity offer promising avenues for GC screening and stratified therapeutic approaches. Research indicates that tRF-23-Q99P9P9NDD is significantly elevated in the serum of GC patients. Specifically, it demonstrates the capability to effectively differentiate GC patients from those with gastritis and healthy donors. Furthermore, its diagnostic efficacy surpasses that of traditional biomarkers such as CEA, CA199, and CA724 [52]. Notably, tRF-17-18VBY9M exhibits specific upregulation in both serum and tissue specimens. Of particular diagnostic significance, this tRF demonstrates exceptional performance in distinguishing early-stage GC from benign lesions, showing remarkable precision [45] (Table 3). Of particular note, serum hsa_tsr016141 has been identified as a novel non-invasive biomarker that shows significant upregulation in GC patients. This discovery substantially expands the potential applications of tRFs in the field of liquid biopsy [70]. Notably, tRF-23-Q99P9P9NDD demonstrated diagnostic efficacy superior to CEA, CA199, and CA724 in the same patient cohort, providing direct head-to-head evidence for its potential advantage over conventional biomarkers [52]. Similarly, tRF-17-18VBY9M exhibited high diagnostic accuracy in distinguishing early-stage GC from benign lesions, with ROC analysis confirming its superior performance compared to traditional markers [45]. These findings, while promising, should be interpreted with caution given the relatively modest sample sizes and lack of multicenter validation in the current literature.

**Table 3:** Potential tRF biomarkers in GC diagnosis

tRF Name	Sample Source	Expression Change	Diagnostic efficiency	References
tRF-23-Q99P9P9NDD	Serum	Upregulated	Effectively distinguishes GC patients from gastritis patients and healthy controls; diagnostic efficacy superior to CEA, CA199, CA724	[52]
tRF-17-18VBY9M	Serum/Tissue	Upregulated	Excellent performance in distinguishing early-stage GC from benign lesions; high diagnostic value as shown by ROC analysis	[45]
hsa_tsr016141	Serum	Upregulated	Novel non-invasive biomarker significantly upregulated in GC patients; expands potential of tRFs in liquid biopsy	[70]
tRF-24-6VR8K09LE9	Tissue	Downregulated	Low expression serves as an early molecular warning marker; delays tumor progression by inhibiting the PI3K/AKT pathway	[53]
tRF-33-P4R8YP9LON4VDP	Plasma	Upregulated	Inhibits tumor via STAT3 pathway; demonstrates clinical value for early detection and prognosis assessment	[44]
tRF-31-U5YKFN8DYDZDD	Serum	Decreased postoperatively	Postoperative decline predicts favorable outcome; potential for prognostic monitoring	[71]
tRF-27-87R8WP9N1E5	Plasma	Upregulated	Positively correlates with tumor size and Ki67 expression; levels decrease postoperatively; useful for early diagnosis, treatment monitoring, and prognosis	[72]

Moreover, accumulating evidence highlights the dual diagnostic and therapeutic potential of specific tRFs in GC through their regulation of key signaling pathways. For example: tRF-24-6VR8K09LE9 inhibits tumor progression by suppressing the PI3K/AKT pathway, with its downregulation serving as a molecular warning marker for early-stage disease [53]; tRF-33-P4R8YP9LON4VDP impedes tumor growth via STAT3 pathway modulation, demonstrating clinical value in both early detection and prognosis [44]. Notably, dynamic tRF expression profiles correlate with treatment response: Postoperative declines in serum tRF-31-U5YKFN8DYDZDD levels predict favorable outcomes [71]; decreasing plasma tRF-27-87R8WP9N1E5 concentrations track with tumor burden reduction, offering a non-invasive metric for therapeutic monitoring and recurrence surveillance [72]. These findings collectively establish tRFs as multifunctional regulators of GC pathogenesis and versatile biomarkers that enhance diagnostic precision while guiding personalized treatment strategies.

#### Advances in tRFs detection technology in GC

Detection technologies for GC-associated tRFs are continuously evolving, providing comprehensive solutions for precision diagnosis. Researchers have screened and validated differentially expressed tRFs in GC tissues and cells using next-generation sequencing combined with qRT-PCR [39], complemented by techniques such as agarose gel electrophoresis, Sanger sequencing, and nucleocytoplasmic RNA separation assays to assess their stability and subcellular localization [70]. For liquid biopsy applications, an absolute quantification method utilizing stem-loop structured reverse transcription primers and TaqMan probes was developed, enabling precise measurement of tRF-27-87R8WP9N1E5 copy numbers in plasma. This biomarker demonstrated significant elevation in GC patients, showing positive correlations with tumor size (*p* < 0.01) and Ki67 expression (*p* < 0.05). Notably, its levels decreased postoperatively, underscoring its integrated value for early diagnosis, treatment efficacy monitoring, and prognostic assessment [72]. It should be noted that the cited study did not report adjustment for multiple comparisons; thus, these p-values are presented as nominal significance levels and warrant confirmation in hypothesis-testing designs with appropriate multiplicity correction. These technological breakthroughs have accelerated the translation of tRFs from basic research to clinical applications, establishing them as reliable tools for non-invasive diagnosis and dynamic monitoring of GC.

#### Potential of tRFs in targeted therapy for GC

The mechanistic diversity of tRFs opens multiple avenues for therapeutic intervention [73]. For tumor-suppressive tRFs (e.g., tRF-Tyr, tRF-33), synthetic mimics could restore their expression and inhibit oncogenic pathways [20, 44, 74]. Conversely, for oncogenic tRFs (e.g., tRF-Val), antisense oligonucleotides or small-molecule inhibitors may block their pro-tumorigenic functions [49, 75]. Notably, the ability of tRFs to modulate immune-related pathways (e.g., TLR/NF-κB) raises the possibility of combining tRF-based therapies with immune checkpoint inhibitors—a strategy that leverages the unique immunomodulatory capacity of tRFs beyond conventional targeted agents [73]. To advance tRF-based therapies toward clinical evaluation, several evidence gaps must be addressed: (1) rigorous preclinical toxicology and pharmacokinetic studies in relevant animal models; (2) optimization of delivery systems tailored to the unique structural features of tRFs; and (3) phase I trials establishing safety and preliminary efficacy in patients with advanced GC. In contrast, miRNA-based therapeutics have already entered clinical trials (e.g., MRX34, cobomarsen), offering valuable lessons for the clinical translation of tRF-based approaches [64].

## Challenges and controversies in tRFs Research

### Functional contradictions: context-dependent roles of tRFs

Conflicting reports on identical tRFs—some promoting apoptosis, others enhancing metabolic adaptation—likely stem from multiple sources of variability rather than true biological paradoxes [75, 76]. First, model system heterogeneity: different GC cell lines (e.g., AGS vs. MKN45) harbor distinct genetic backgrounds and baseline signaling activities, which can dramatically alter tRF functionality. Second, tRF nomenclature and isotype resolution: the same name may encompass molecules derived from different tRNA isodecoder genes or carrying distinct post-transcriptional modifications (e.g., m⁵C, m⁷G, pseudouridine), which profoundly influence protein binding and biological activity [77–80]. Third, detection technology and bioinformatic pipelines: variability in RNA-seq library preparation, sequencing depth, and alignment strategies yields inconsistent tRF profiles across studies. Fourth, tissue-plasma dissociation: a tRF upregulated in plasma (e.g., tRF-33-P4R8YP9LON4VDP) may function as a tumor suppressor in tissue-based assays, reflecting active secretion rather than intracellular oncogenic function [42, 44]. Future studies using matched tissue and liquid biopsy samples are essential to disentangle these relationships.

### Translational challenges: beyond the usual ncRNA hurdles

While tRFs share certain barriers with other oligonucleotide therapeutics, their unique features present distinct challenges. Delivery specificity requires custom-designed vehicles—such as lipid nanoparticles, exosome-based platforms, or tumor microenvironment-responsive carriers—to ensure precise tumor localization, yet each faces hurdles in tissue selectivity and endosomal escape [81, 82]. Although tRNA modifications confer intrinsic stability, therapeutic applications demand further enhancement through chemical modifications (e.g., phosphorothioate backbones, 2’-O-methylation, locked nucleic acids) while balancing the risk of immunogenicity, particularly TLR receptor activation [83, 84]. Finally, the substantial inter-patient heterogeneity in tRF expression—influenced by genetic background, tumor stage, and epigenetic characteristics—necessitates personalized approaches; large-scale multicenter cohorts are required to establish robust correlations between tRF molecular subtypes and treatment responses [85, 86].

### The evidence gap: from discovery to clinical utility

A fundamental gap exists between current preclinical findings and the level of evidence needed for clinical adoption. A search of ClinicalTrials.gov reveals no registered trials evaluating tRF-based diagnostics or therapeutics in GC. To bridge this gap, future studies must prioritize: (1) analytical validation—standardized assays with defined performance characteristics; (2) clinical validation—large prospective cohorts with blinded outcome assessment and direct comparison to established biomarkers; and (3) demonstration of clinical utility—evidence that tRF-based approaches improve patient outcomes compared to current standard of care. These requirements, though not unique to tRFs, are often underappreciated in early discovery research.

### Toward resolution: a call for methodological standardization

Addressing these controversies requires concerted efforts to establish standardized frameworks for tRF research. Key priorities include: adopting uniform nomenclature that specifies tRNA isotype, cleavage site, and modification status; developing community-accepted bioinformatic pipelines for tRF identification and quantification; requiring experimental validation in multiple cell lines and, where feasible, patient-derived organoids or xenografts; promoting data sharing of raw sequencing files to enable cross-study meta-analyses; and establishing reporting guidelines analogous to MIAME or MIQE. Until such standardization is achieved, conflicting findings will persist, and the translational potential of tRFs will remain incompletely realized.

## Conclusion and prospects

In this review, we have systematically examined the emerging landscape of tRNA-derived fragments in GC through a comparative lens, highlighting their distinctive biological features and translational potential relative to other non-coding RNAs. tRFs exhibit a uniquely versatile mechanistic repertoire that extends beyond canonical miRNA-like gene silencing to include direct modulation of RNA-binding proteins such as EEF1A1 and hnRNPD. This functional diversity, coupled with their inherent stability and tissue-specific expression patterns, positions tRFs as promising candidates for both diagnostic biomarkers and therapeutic interventions.

The diagnostic potential of tRFs is particularly noteworthy. Multiple candidates have demonstrated superior performance to conventional biomarkers in head-to-head comparisons, with the ability to distinguish early-stage GC from benign lesions. However, these promising findings must be tempered by a realistic appraisal of the substantial translational barriers that remain—including functional heterogeneity, detection standardization challenges, and the complete absence of registered clinical trials to date.

Looking forward, several strategic directions merit prioritization. First, the field urgently requires methodological standardization, uniform nomenclature, community-accepted bioinformatic pipelines, and reporting guidelines analogous to MIAME or MIQE — essential prerequisites for reproducible progress. Second, rigorous clinical validation in large, multicenter cohorts with direct comparison to established biomarkers will be necessary to establish clinical utility. Third, deeper mechanistic dissection of tRF biology, including the role of post-transcriptional modifications, subcellular localization dynamics, and context-dependent functions, will inform rational therapeutic design.

The application of single-cell and spatial transcriptomics to tRF research remains nascent and requires technical adaptation. Conventional single-cell platforms have limited capture efficiency for short non-coding RNAs; therefore, future efforts should develop small-RNA-optimized sequencing protocols (e.g., Smart-seq-total or pre-tagmentation-based methods) to enable accurate profiling of tRF expression across distinct gastric cancer cell subsets. In parallel, spatial transcriptomics—combined with customized probes or in situ hybridization strategies—could localize tRFs within tissue sections, revealing their spatial heterogeneity at tumor invasion fronts, immune infiltration zones, and across histological subtypes (diffuse vs. intestinal). Once matured, these technologies will propel tRF research from the averaged perspective of bulk tissue homogenates toward a cell-resolved, spatially resolved resolution, offering new dimensions for understanding tRF functional networks within the gastric cancer microenvironment. Together with engineered delivery systems (e.g., targeted exosomes, tumor-responsive nanoparticles), these advances will help bridge the critical gap from mechanistic discovery to clinical translation [87–90]. Ultimately, realizing the translational potential of tRFs will require sustained interdisciplinary collaboration across molecular biology, bioinformatics, materials science, and clinical medicine—moving the field beyond descriptive studies toward transformative strategies that address the unmet needs of GC patients.

## Data Availability

N/A.

## Declarations

### Declaration

Generative AI and AI-assisted technologies were NOT used in the preparation of this work.

## Abbreviations

tRFs: tRNA-derived fragments
GC: Gastric cancer
ncRNAs: Non-coding RNAs
Ago: Argonaute
TLR/NF-κb: Toll-like receptor/nuclear factor kappa B
tsRNAs: tRNA-derived small RNAs
tiRNAs: tRNA halves
i-tRFs: Internal tRNA-derived fragments
OB: Oligonucleotide/oligosaccharide-binding
RBPs: RNA-binding proteins
STAT3: Signal transducer and activator of transcription 3
EEF1A1: Eukaryotic translation elongation factor 1 alpha 1
MDM2: Mouse double minute 2 homolog
hnRNPD: Heterogeneous nuclear ribonucleoprotein D
TTK: Threonine tyrosine kinase
ROC: Receiver operating characteristic
tRFdb: tRNA-derived fragments database
